# Ectopic Pinna

**DOI:** 10.1016/S1808-8694(15)30534-6

**Published:** 2015-10-18

**Authors:** P.N. Tejankar, Mudit Mittal

**Affiliations:** 1 Ectopic Pinna (senior Otolaryngologist); 2 Ectopic Pinna (resident surgical officer)

**Keywords:** ectopic pinna

## INTRODUCTION

Ectopic Pinna is rare presentation. One very rare case of Ectopic Pinna is reported here, where the whole of the Pinna was located close to Lateral Canthus of Right Eye.

Anomalies of the external ear may occur in isolation or associated with middle or inner ear or may be a part of wide spread syndrome. There may be Anotia, Microtia, Macrotia, Polyotic, Synotia, Melotia, Abnormalities of lobule and Abnormalities of helix etc.^1^

## CASE REPORT

A 21/2 year old Male child was brought by his attendants to our clinic with abnormal position of Right Pinna. On examination Right Pinna was seen displaced towards the lateral canthus of the Right eye. The External Auditory canal was at its position. But Tympanic Membrane was not seen on Otoscopy due to narrowing of the canal as well as patient was not co-operative. A tubercle was seen over the Right Mastoid region near the External Auditory Canal. A tag of skin was also noticed attached to Right lateral canthus of eye. Patient's Left ear was within normal limit. A Hemangioma was also seen over left arm near axilla as incidental finding. The nose and the throat were normal and nothing specific was found. A detailed history of the patient was taken including the Antenatal, Natal and Postnatal details. The patient was normal full term hospital delivery and nothing significant was found in history. Patient was advised complete checkup by Pediatrician to rule out any congenital anomaly and C.T. Scan Temporal bone to see the Ossicular Chain status, any aberrant course of facial nerve and to rule out other associated congenital deformities. Patient was also advised to get his hearing assessment done, but patient left the clinic against medical advice.

**Figure 1:**
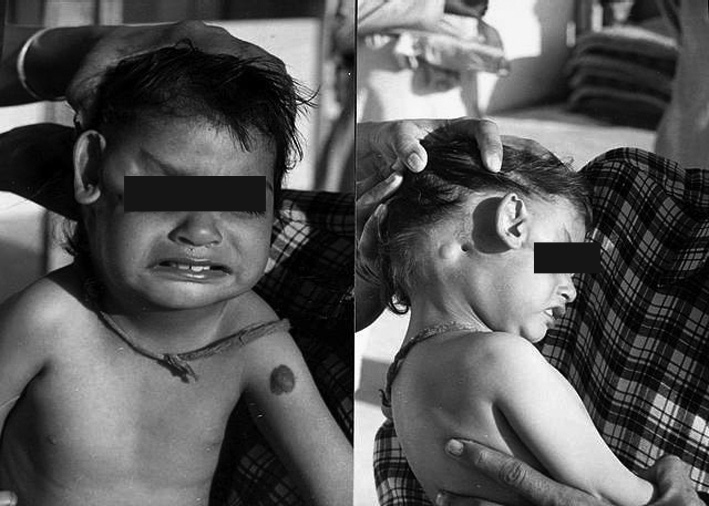
Ectopic Pinna - Right Pinna, displaced towards the lateral canthus of the Right eye. A tubercle present over the Right Mastoid region near the External Auditory Canal. A Hemangioma over left arm. F

## DISCUSSION

The development of the auricle begins with the appearance of six hillocks around the first pharyngeal groove between the first and the second arches. Three hillocks develop on the same side of the grove; but as growth proceeds they tend to become obscured and of those of the first (Mandibular) arch, only that which later forms the Tragus can be obviously identified throughout the process. It seems that the bulk of the auricle is derived from the mesenchyme of the second (hyoid) arch.^2^

Congenital abnormalities of the ear can be shown in great details by CT if there is osseous deformity but MRI is now providing evidence of soft tissue lesions in some cases. Structural abnormalities of the inner, middle and external ear can be shown in considerable detail by Tomographic techniques. Congenital deformities of middle and external ear are seen much more common than deformities of inner ear, although combined deformities occurs in about 20% of the patients.^3^

## FINAL COMMENTS

1. The External Ear deformities are usually associated with deformities of middle and internal ear.
2. CT scan is the investigation, which can give a good picture of associated middle and inner ear anomalies, Ossicular chain continuity and aberrant course of facial nerve with external ear deformities.
3. Management of this patient needs combined surgical efforts of an ENT surgeon and Plastic surgeon in stages with strict follow up.
